# Should we avoid protease inhibitors in first lines due to their lipid side effects? - cons

**DOI:** 10.1186/1471-2334-14-S2-S12

**Published:** 2014-05-23

**Authors:** Hans-Jürgen Stellbrink

**Affiliations:** 1 Hospital of Hamburg, Infectious Diseases Department, Hamburg, Germany

## 

Treatment is medical risk management. In treatment decisions, the relative risks of interventions have to be weighed against each other, with long-term irreversible untoward events being most important to avoid. Resistance development is such a deleterious long-term effect, especially with lower adherence, as it reduces the numbers of options for successful long-term viral suppression. It is less frequent with PIs, even with lower levels of adherence. Therefore, PIs are often used to in order to prevent resistance development in settings in which it may otherwise arise. They represent an attractive option for avoiding exposure to other drug classes with their associated toxicities, especially NRTIs, and thus are the backbone of most currently investigated alternative regimens. Lipid elevations are common side effects of protease inhibitor therapy (PIs). Their relevance, however, lies in their association with clinical events. Not all PIs lead to the same extent of lipid increase, and not all are associated with myocardial infarction and stroke. Lipid elevations are reversible after switching to other regimens, and the associated risk is time-dependent, so that there remains time to manage cardiovascular risk. Moreover, in the complex array of cardiovascular risk factors, lipid elevations are just one factor, with their relative contribution to risk increasing with age, and the absolute number of events being low at younger ages. There are numerous promising interventions for reducing cardiovascular risk substantially (e.g. smoking cessation, physical activity, statins), so that avoiding lipid elevations due to PIs should not be the primary concern. Long-term viral suppression still remains the most important consideration for selecting antiretroviral drugs, as uncontrolled HIV replication remains the major threat to long-term health of an infected person. Therefore, despite a lot of other options, the high genetic barrier to resistance of PIs reserves them a place in first-line therapy for many patients.

